# Opposite modulation of brain stimulation reward by NMDA and AMPA receptors in the ventral tegmental area

**DOI:** 10.3389/fnsys.2013.00057

**Published:** 2013-10-03

**Authors:** Charles Ducrot, Emmanuel Fortier, Claude Bouchard, Pierre-Paul Rompré

**Affiliations:** ^1^Département de Physiologie, Université de MontréalMontréal, QC, Canada; ^2^Département de Psychiatrie, Université de MontréalMontreal, QC, Canada; ^3^FRSQ Research Group in Behavioral Neurobiology, Department of Psychology, Concordia UniversityMontréal, QC, Canada

**Keywords:** AMPA, glutamate, NMDA, reward, ventral midbrain

## Abstract

Previous studies have shown that blockade of ventral tegmental area (VTA) glutamate N-Methyl-D-Aspartate (NMDA) receptors induces reward, stimulates forward locomotion and enhances brain stimulation reward. Glutamate induces two types of excitatory response on VTA neurons, a fast and short lasting depolarization mediated by α-amino-3-hydroxy-5-methyl-4-isoxazole propionate (AMPA) receptors and a longer lasting depolarization mediated by NMDA receptors. A role for the two glutamate receptors in modulation of VTA neuronal activity is evidenced by the functional change in AMPA and NMDA synaptic responses that result from repeated exposure to reward. Since both receptors contribute to the action of glutamate on VTA neuronal activity, we studied the effects of VTA AMPA and NMDA receptor blockade on reward induced by electrical brain stimulation. Experiments were performed on rats trained to self-administer electrical pulses in the medial posterior mesencephalon. Reward thresholds were measured with the curve-shift paradigm before and for 2 h after bilateral VTA microinjections of the AMPA antagonist, NBQX (2,3,-Dioxo-6-nitro-1,2,3,4-tetrahydrobenzo(f)quinoxaline-7-sulfonamide, 0, 80, and 800 pmol/0.5 μl/side) and of a single dose (0.825 nmol/0.5 μl/side) of the NMDA antagonist, PPPA (2*R*,4*S*)-4-(3-Phosphonopropyl)-2-piperidinecarboxylic acid). NBQX produced a dose-dependent increase in reward threshold with no significant change in maximum rate of responding. Whereas PPPA injected at the same VTA sites produced a significant time dependent decrease in reward threshold and increase in maximum rate of responding. We found a negative correlation between the magnitude of the attenuation effect of NBQX and the enhancement effect of PPPA; moreover, NBQX and PPPA were most effective when injected, respectively, into the anterior and posterior VTA. These results suggest that glutamate acts on different receptor sub-types, most likely located on different VTA neurons, to modulate reward.

## Introduction

The ventral tegmental area (VTA) contains dopamine neurons that give rise to the ascending mesocorticolimbic pathway that plays a key role in motivation and reward. A large body of evidence shows that dopamine neurons are activated by stimuli that have a positive reinforcing property. Drugs of abuse, for instance, stimulate dopamine cell firing and/or increase synaptic dopamine levels in the nucleus accumbens (Wise and Rompré, 1989; Wise, 1996), a limbic region considered as a functional interface between motivation and action (Mogenson et al., 1980). Reward induced by electrical brain stimulation also increases dopamine cell firing and accumbens dopamine release (Moisan and Rompré, 1998; Hernandez and Shizgal, 2009). But dopamine neurons do not respond uniquely to appetitive stimuli. Brishoux et al., (2009) found a sub-group of dopamine neurons that was activated by footshock. Activation of different dopamine sub-populations by stimuli that have opposite motivational valence is likely mediated by different VTA afferent inputs. Ventral midbrain neurons are under the control of numerous glutamatergic afferents originating from cortical and subcortical limbic regions (Carr and Sesack, 2000; Geisler et al., 2007; Omelchenko et al., 2009) and from VTA interneurons (Dobi et al., 2010). Selective activation of VTA glutamatergic afferent inputs from the laterodorsal tegmentum nucleus induces conditioned-place preference while activation of the lateral habenula glutamatergic inputs produces conditioned-place aversion, opposite motivational effects that appear to be mediated by activation of the mesoaccumbens and mesoprefrontal dopamine pathway respectively (Lammel et al., 2012). The rewarding value of a stimulus is signaled when firing of dopamine neurons shifts from a tonic to a phasic mode, and this mode of firing is associated with enhanced dopamine release (Gonon, 1988; Schultz, 2007). The induction of burst firing pattern of dopamine neurons is under the control of glutamatergic afferents from the laterodorsal tegmental nucleus (Lodge and Grace, 2006). Consistently, Lammel et al., (2012) showed that activation of mesoaccumbens dopamine neurons by laterodorsal tegmental afferents was blocked by the AMPA antagonist, CNQX, and the conditioned-placed preference prevented by blockade of accumbens D1/D2- like dopamine receptors. But the modulatory role of glutamate is not limited to dopamine neurons. Glutamatergic terminals from efferent neurons of different limbic nuclei and from local interneurons establish synaptic connections with non-dopamine neurons (Carr and Sesack, 2000; Omelchenko et al., 2009; Dobi et al., 2010). Among these non-dopamine neurons are GABAergic neurons that exert a negative modulation on dopamine activity (Grace et al., 2007). Selective activation of lateral habenula glutamatergic pathway induces excitatory post-synaptic currents (EPSCs) in GABAergic neurons located in the rostromedial tegmental nucleus also named the tailed of VTA (RMtg/tVTA; Lammel et al., 2012). These neurons send an inhibitory input to more rostral VTA dopamine neurons and this signal is triggered by aversive stimuli (see Bourdy and Barrot, 2012). It appears then that glutamate can generate opposite motivational effects by acting either on different VTA dopamine and/or GABA neurons. It is not clear at this point whether these different motivational effects are mediated by different glutamatergic receptors. Both activation and blockade of VTA NMDA receptors increases accumbens dopamine release (French et al., 1993; Karreman et al., 1996; Mathe et al., 1998; Kretschmer, 1999) and stimulates forward locomotion (Kretschmer, 1999; Cornish et al., 2001). Mice (David et al., 1998) and rats (Webb et al., 2012) can be trained to self-administer the NMDA antagonists, AP-5 and AP-7, into the VTA, suggesting that a positive motivational valence predominates following blockade of the receptors. Consistently, Bergeron and Rompré, (2013) found that blockade of VTA NMDA receptors enhances brain stimulation reward and operant responding, effects that are mostly likely mediated by NMDA receptors that are devoid of GluN2b subunits. Less is known about the role of VTA AMPA receptors in reward. Activation of VTA AMPA receptors stimulates dopamine impulse flow and accumbens dopamine release (Chergui et al., 1993; Karreman et al., 1996). But blockade of VTA AMPA receptors stimulates locomotor activity, increases operant responding for conditioned reinforcement and induces conditioned-place preference, behaviors that reflect of a positive motivational effect (Harris and Aston-Jones, 2003; Harris et al., 2004; Nolan et al., 2010).

To further clarify the role of VTA AMPA receptors in reward, we investigated the effects of VTA microinjections of the AMPA antagonist, NBQX, on reward and operant responding induced by electrical brain stimulation. We also tested the effects of PPPA, a NMDA antagonist, to determine whether blockade of each receptor sub-type independently at the same VTA sites produces similar effects.

## Experimental procedures

### Animals

Male Long-Evans rats (Charles River Canada) weighting between 300 and 350 g at the time of surgery were used. They were housed 2 per cage (1 per cage after surgery) in a temperature (22°C) and humidity (40%) controlled room with a 12 h light/dark cycle (lights on at 06:00). They were allowed to habituate for 7 days to the housing environment before surgery and had free access to food and water. All procedures were in accordance with the guidelines of the Canadian Council on Animal Care and all efforts were made to minimize suffering and the number of animals used.

### Surgery

Rats were anaesthetized with isoflurane (2.5–3.5%, O_2_ 0.7 L/min) and placed on a stereotaxic apparatus. The surface of the skull was exposed between lambda and bregma and burr holes were made into the cranium at the point of insertion of the stimulation electrode and the guide cannulae. A moveable stimulation electrode (Miliaressis, 1981) made from a 0.27 mm stainless steel wire insulated with epoxy (except for the round tip) was implanted into the postero-medial mesencephalon using the following flat skull coordinates: 7.6 mm posterior to bregma, 0.0 mm lateral to the midline and 6.8 mm below the surface of the skull (Paxinos and Watson, 1986). Because electrical stimulation of this area activates reward-relevant axons that travel bilaterally through the VTA (Boye and Rompré, 1996), a guide cannula (HRS Scientific, Montreal, Canada, model C315G) was implanted in each hemisphere; coordinates were 5.4–5.6 mm posterior to bregma, 3.2 mm lateral to the midline (18° medio-lateral angle) and 6.4 mm below the surface of the skull; each cannula was closed with an obturator of the same length. A bare wire connected to a male Amphenol connector was wrapped around four stainless steel screws that were threaded into the skull; it served as the inactive electrode. The cannula/electrode assembly was anchored to the skull with dental acrylic. A 0.05 mL injection of Duplocillin LA containing 15,000 I.U. of penicillin was administered (im) to prevent infections. The analgesic Anaphen (5 mg/kg, sc) was administered at the end of surgery.

### Behavioral training

Five to seven days after surgery, rats were placed in a test cage (25 × 25 cm) made from polymer walls and one front Plexiglas wall that allowed observation. To reduce disturbance from external noise, test cages were encased in ventilated melamine boxes. Each test cage was equipped with an infrared photocell inside a hole (3 cm diameter and 3 cm deep) located 2 cm above the wire-mesh floor. Interruption of the photocell triggered a constant-current pulse generator (Mundl, 1980) that delivered a single 400 ms train of 0.1 ms cathodal rectangular pulses. Each train was followed by a period of 600 ms during which the pulse generator could not be triggered (see Bergeron and Rompré, 2013). Using the standard shaping procedure, rats were trained to produce a nose poke to receive a train of stimulation; the current and the frequency were varied until the rat learned the task. If a rat did not learn to respond, the electrode was lowered by 0.2 or 0.4 mm and a new site was tested; the electrode was lowered this way until the rat learned to respond regularly. Following this period of shaping, rats were trained to respond during discrete 55-s trials, each being followed by an interval of 15-s during which stimulation was not available. The beginning of each trial was signaled by 5 trains of non-contingent priming stimulation delivered at a rate of 1 per second. With the current intensity held constant, the frequency (number of pulses per train * 2.5) was varied from 100 to 30 Hz in 0.06–0.09 log unit steps; this generated a curve relating the number of nose-pokes per trial to the stimulation frequency (rate/frequency or R/F curve). An index of reward threshold was inferred from each R/F curve and it was defined as the pulse frequency sustaining a half-maximal rate of responding (M50). The current intensity was set for each rat to generate a M50 value between 60 and 70 Hz. During the training phase, four R/F curves were determined during consecutive daily test sessions. Testing began when the lower and higher M50 values derived from the last three R/F curves varied by less than 0.1 log unit for 3 days.

### Drug and vehicle tests

A first saline test was carried out to habituate the animals to the injection procedure. This test was similar to the subsequent drug and vehicle tests and consisted of determining four R/F curves before and seven curves after the injections. Bilateral injections were made by inserting into each guide cannula an injection cannula (model C315I) that extended 2 mm beyond the tip of the guide. Each injection cannula was connected with polyethylene tubing to a 2-μl microsyringe and a 0.5 μl volume of sterile 0.9% saline was injected into each hemisphere simultaneously with a micro-infusion pump over a period of 60 s; the injection cannulae were left in place for an additional 60 s to allow diffusion into the tissue. Results from this test were not included in the analysis. Drug and vehicle tests began at least 5 days after this first habituation test. On a test day, four baseline R/F curves were first determined over a period of 70 min; the first one was considered as a warm-up and discarded. Then each rat was centrally injected with the drug, or its vehicle, using the procedure described above and seven additional R/F curves were determined over a test session that lasted approximately 125 min, starting immediately after the injection. Rats were initially tested with one of two doses of NBQX [80 (30 ng) and 800 (300 ng) pmol/0.5μl/side], or the vehicle; they received all doses (including vehicle) in a counterbalanced order. These doses were based on previous studies (Nolan et al., 2010; Waraczynski et al., 2012) and on preliminary data from our lab. Following completion of the NBQX tests, we tested the effect of a single dose of the NMDA antagonist, PPPA, (0.825 nmol/0.5 μl/side), that was previously reported to alter reward (Bergeron and Rompré, 2013). There were at least 7 days between two consecutive drug or vehicle tests.

### Data analysis

The mean changes in M50 (reward threshold) and maximal rate of responding (maximal rate of nose poke from each R/F curve) were expressed as the percentage of pre-injection value (baseline) and group means were calculated for each dose and vehicle result. Mean percentage change of both M50 and maximum response were analyzed with a Two-Way (dose × time) analysis of variance (ANOVA) for repeated measures. Homogeneity of variance was tested and square root or log data transformations were performed or Greenhouse-Geiser correction of degrees of freedom used when necessary. Comparisons among means were made with the Holm-Sidak test with the level of significance set at 0.05 (SigmaStat, V11.0, Systat Software Inc.; IBM SPSS Version 20).

### Histology

At the end of the experiment, the animals were deeply anesthetized with urethane (1.4–2.0 g/kg, i-p.) and the stimulation and injection sites were marked by passing an anodal current of 0.1 mA during 60 s through the electrode and the two injection cannulae that were inserted into the guides. The animals were then perfused with 0.9% saline followed by a 10% formalin solution and the brains were extracted and soaked in a solution containing 3% potassium ferrocyanide, 3% potassium ferricyanide and 0.5% trichloroacetic acid for 24 h. The brains were then rinsed and stored in a 10% formalin solution for several days. They were subsequently frozen and sliced in 40 μm sections that were mounted on gelatin-coated glass slides. The location of the stimulation and of the injections sites were determined under light microscopy from freshly sliced sections and/or sections stained with Nissl's technique. Animals that had both injection sites within the VTA, including the rostral and caudal linear nuclei, the paranigral, parabrachial and the interfascicular nuclei, and the medial part of the substantia nigra between 4.8 and 6.0 mm behind bregma (Paxinos and Watson, 1986) were included in the analyses.

### Drugs

NBQX disodium (2,3,-Dioxo-6-nitro-1,2,3,4-tetrahydrobenzo(f)quinoxaline-7-sulfonamide disodium), and PPPA [(2R, 4S)-4-(3-Phosphopropyl)-2-piperidinecarboxylic acid], were purchased from *Tocris Bioscience* (Ellisville, MI, USA). They were dissolved in sterile 0.9% saline and stored frozen in 20 μl aliquots. Drug solutions were thawed and diluted when necessary just prior to testing. Doses are expressed as salt.

## Results

**Figure 1:**
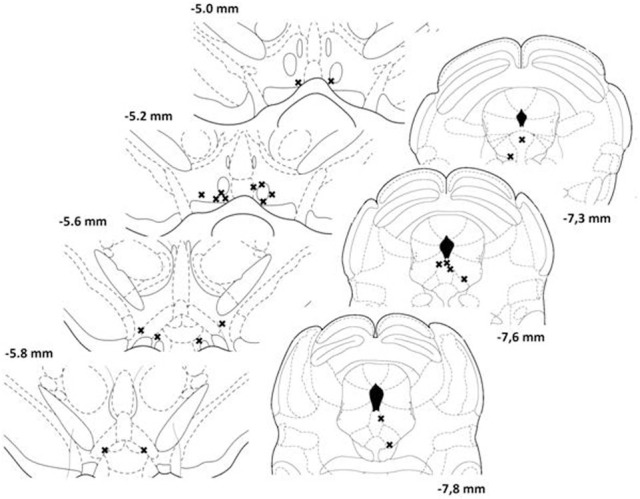
**Location of injection (left panels) and stimulation (right panels) sites for each animal included in the study.** Each site is represented by a single cross. Illustrations are modified drawings from Paxinos and Watson's atlas (1986); the number on the side of each plate represents the anterior-posterior distance (mm) from bregma.

Of the 12 animals initially prepared for the study, 8 were successfully trained and completed the experiment. Histological analysis revealed that the stimulation sites were located within the postero-medial mesencephalon, within the ventral central gray, between the anterior-posterior regions corresponding to 7.3 and 7.8 mm posterior to bregma (Figure 1, right panels). The injection sites for the eight animals included in the analyses are shown in the left panels of Figure 1. Sites were located between 5.0 and 5.8 mm posterior to bregma, within the ventral part of the VTA, a region that contains neurons activated by rewarding electrical stimulation (Moisan and Rompré, 1998; Marcangione and Rompré, 2008).

## NBQX attenuated brain stimulation reward

**Figure 2:**
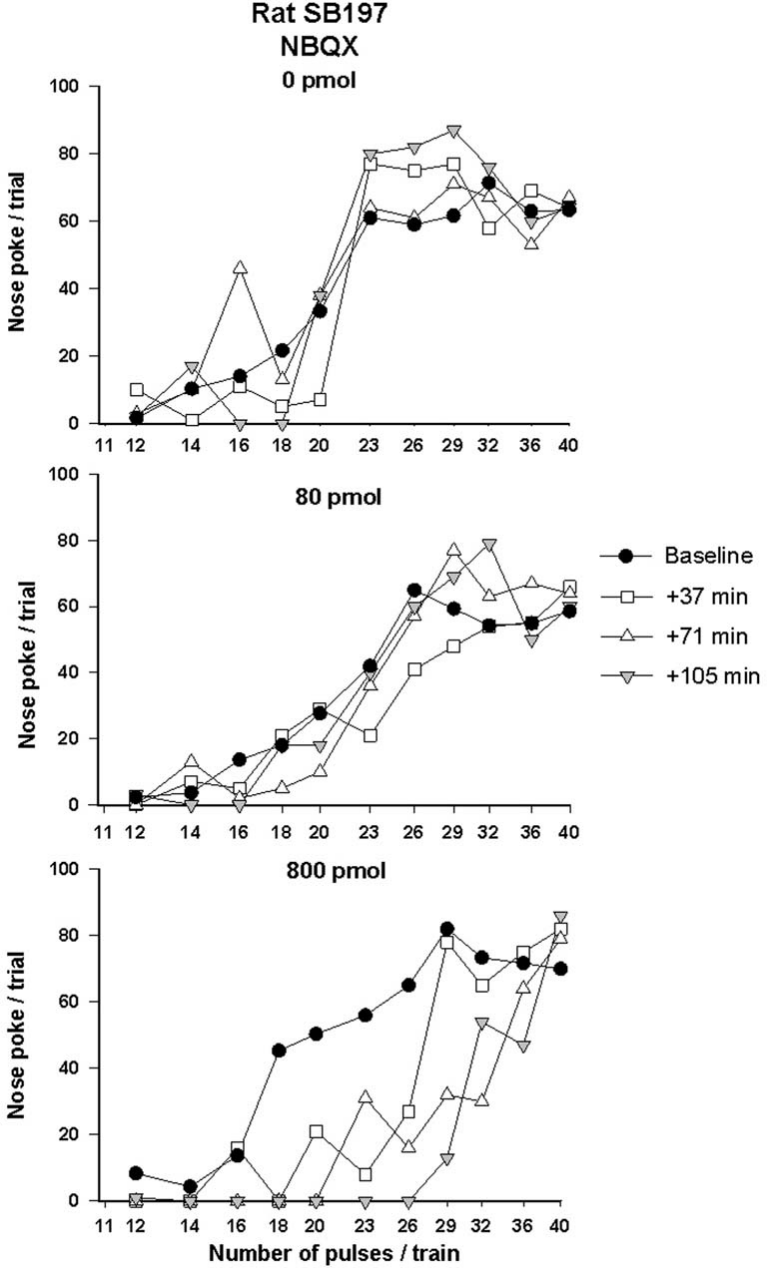
**Rate-frequency curves obtained from rat SB197 prior to (baseline) and after injection of vehicle (top panel, 0 nmol) and two doses (80 and 800 pmol/0.5 μl/side, middle and bottom panels respectively) of NBQX.** For clarity only four curves, including averaged baseline curves, are shown. The *x*-axis represents the number of pulses per train on a log scale.

**Figure 3:**
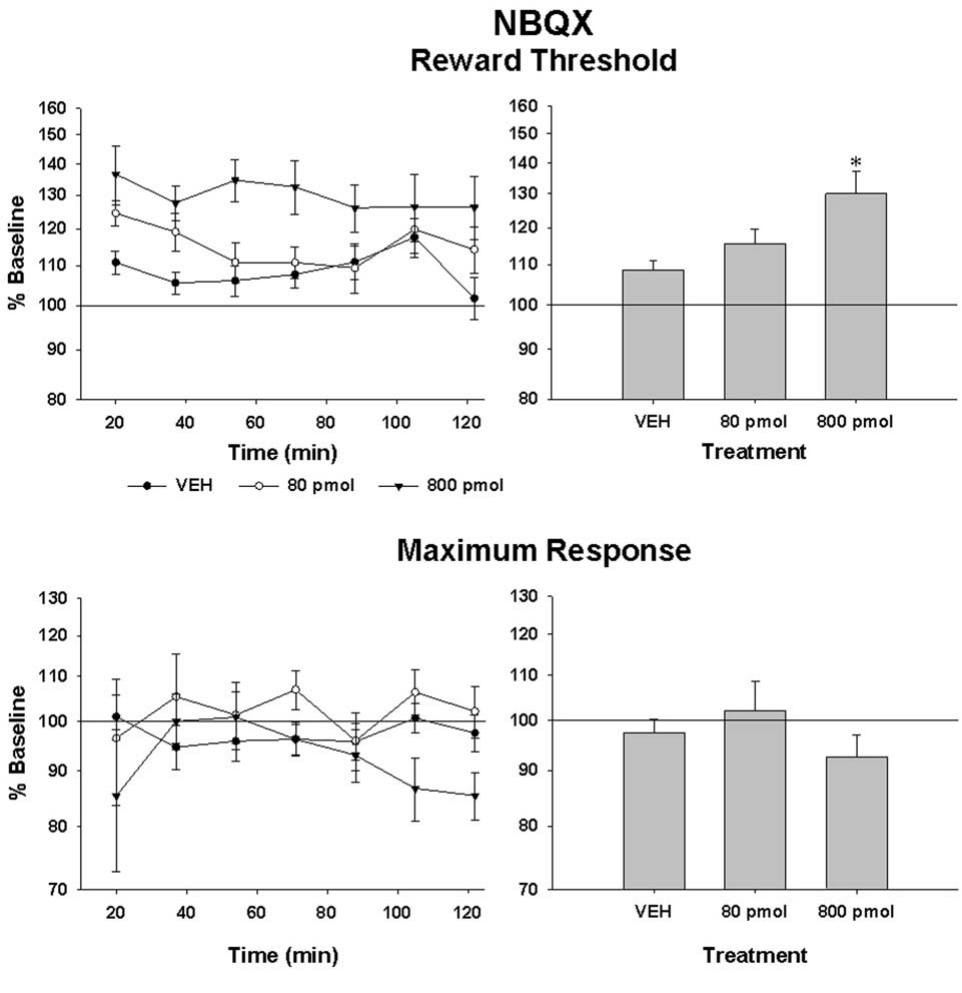
**Changes in reward threshold (top panels) and maximum response (bottom panels) as a function of time (left panels) after injection of vehicle (VEH) and each of the two doses of NBQX.** Data represent the mean (±sem) from eight animals and are expressed as percentage of baseline on a log scale. Changes (mean ± sem) measured over the entire test session are shown in the right panels. Asterisk indicates a statistically significant difference (*p* < 0.05) with VEH.

Rate-frequency curves obtained from rat SB197 after bilateral VTA injections of vehicle, 80 and 800 pmol/0.5 μl/side of NBQX are shown in Figure 2. NBQX produced a dose-dependent rightward shift of the R/F curve reflecting a reduction of the rewarding effectiveness of the stimulation; the larger shift was produced by the high dose. This attenuation of reward was not evident following injections of the vehicle (top panel). Figure 3 shows mean changes (expressed in % of baseline) in reward threshold (top panels) and maximal response (bottom panels) for each dose of NBQX. The magnitude of the increase in reward threshold produced by NBQX varied with the dose but not the time post-injection. Analysis of variance yielded a significant effect of treatment [*F*_(2, 21)_ = 4.92, *p* < 0.05] but no treatment by time interaction [*F*_(12, 126)_ = 1.32, *p* > 0.05]. *Post-hoc* test confirmed that the increase in threshold measured after injection of 800 pmol of NBQX is significantly different than that measured after vehicle; there is no significant difference between vehicle and 80 pmol. Maximum rate of responding was not significantly altered by NBQX (bottom panel). Analysis of variance yielded no significant effect of treatment [*F*_(2, 21)_ = 0.97, *p* > 0.05] and no treatment by time interaction [*F*_(4.755, 49.923)_ = 1.31, *p* > 0.05], suggesting that the increase in reward threshold results from a selective reduction in the rewarding effectiveness of the stimulation.

## PPPA enhanced brain stimulation reward

**Figure 4:**
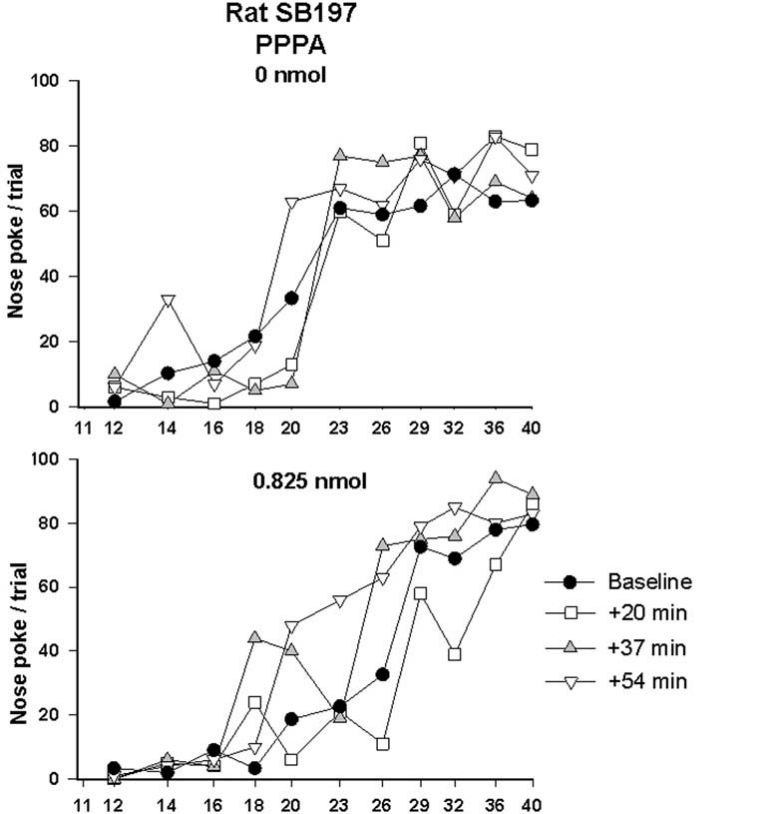
**Rate-frequency curves obtained from rat SB197 prior to (baseline) and injection of vehicle (top panel, 0 nmol) and PPPA (0.825 nmol/0.5 μl/side).** For clarity only four curves, including averaged baseline curves, are shown. The *x*-axis represents the number of pulses per train on a log scale.

**Figure 5:**
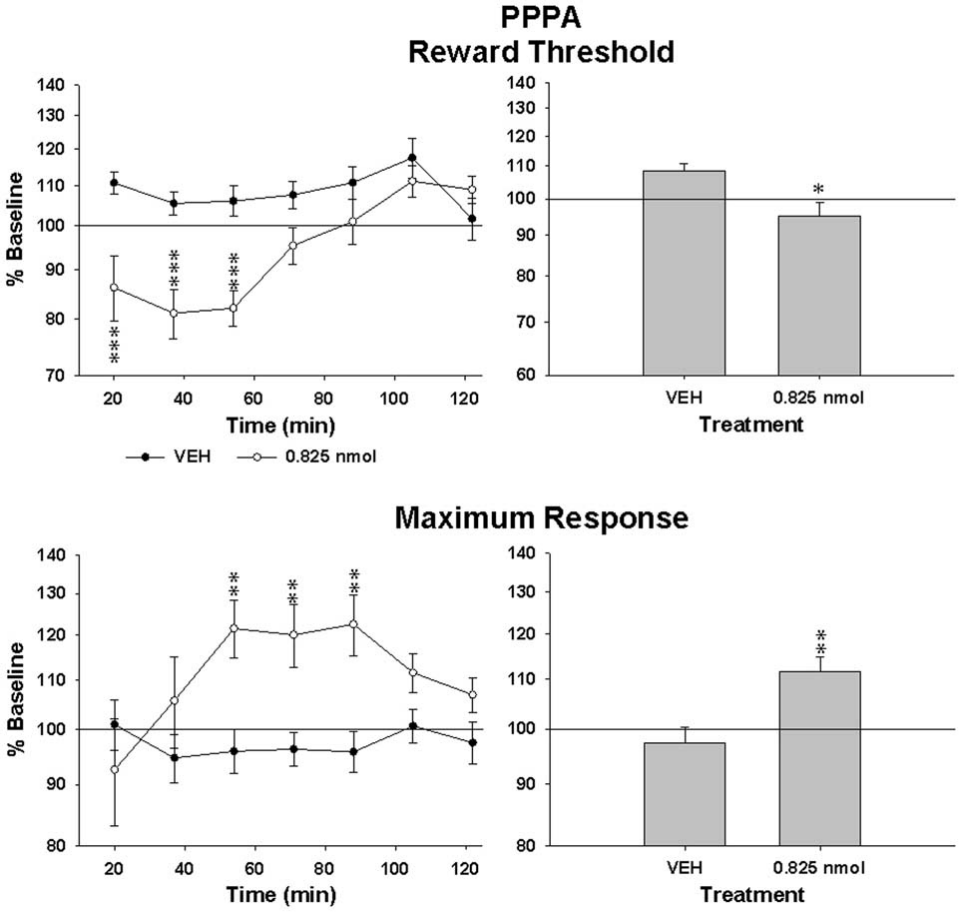
**Changes in reward threshold (top panels) and maximum response (bottom panels) as a function of time (left panels) after injection of vehicle (VEH) and PPPA (0.825 nmol/0.5 μl/side).** Data represent mean (±sem) from eight animals and are expressed as percentage of baseline on a log scale. Changes (mean ± sem) measured over the entire test session are shown in the right panels. Asterisk indicates a significant difference (^*^p < 0.05, ^**^p < 0.01, ^***^p < 0.001) with VEH.

Since AMPA and NMDA receptors can be functionally linked and that both are involved in the modulation of VTA neurons by glutamate, we tested the effect of PPPA, a NMDA antagonist, injected at the same sites in every animal that was tested with NBQX. The dose of PPPA used was found previously to enhance reward when injected into the VTA (Bergeron and Rompré, 2013). Figure 4 illustrates the R/F curves obtained from the same rat, SB197, after bilateral VTA injections of PPPA (bottom panel). PPPA produced initially a small rightward shift of R/F curve that was followed by a shift to the left; the magnitude of the leftward shifts induced by the vehicle was smaller, suggesting that in this rat PPPA slightly enhanced reward. Grouped means from the eight animals tested confirmed the enhancement effect of PPPA (Figure 5, top panels). The ANOVA performed on mean changes in reward threshold yielded a significant effect of treatment [*F*_(1, 14)_ = 8.68, *P* < 0.05] and a significant treatment by time interaction [*F*_(6, 84)_ = 6.45, *p* < 0.001]. *Post-hoc* test showed that PPPA significantly reduced threshold compared to vehicle during the first 54 min post-injection (top, left panel). Unlike NBQX, PPPA produced a significant change in maximum response (bottom panels). The ANOVA yielded a significant effect of treatment [*F*_(1, 14)_ = 9.5, *p* < 0.002] and a significant treatment by time interaction [*F*_(6, 84)_ = 2.8, *p* < 0.02]. *Post-hoc* test showed that the maximum response was enhanced compared to vehicle between 54 and 90 min post-injection. It is interesting to notice the discrepancy between the time course of the effect of PPPA on reward threshold and on maximum response.

## Inverse relationship between the attenuation effect of NBQX and the enhancement effect of PPPA on reward: a site dependent effect

**Figure 6:**
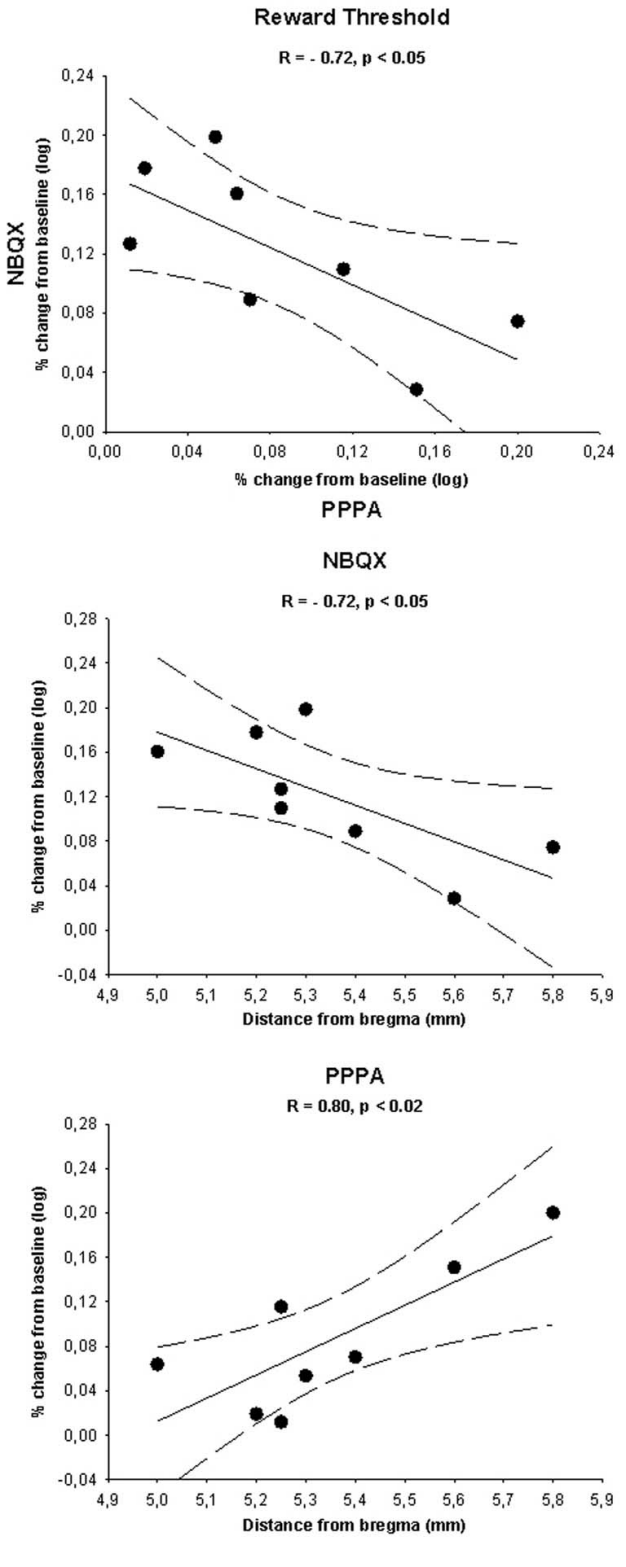
**The top panel shows a scatter plot illustrating the log percent change (from baseline) in reward threshold measured for each rat after injection of 0.825 nmol of PPPA (*x*-axis) and 800 pmol of NBQX (*y*-axis).** Scatter plots illustrating the log percent change (from baseline) in reward threshold measured for each rat after injection of 0.825 nmol of PPPA and 800 pmol of NBQX as a function of the location of the injections sites along the antero-posterior axis of the brain are shown in the middle and bottom panels respectively. Correlation index (R) and level of significance are shown along with the linear regression line (plain line) and the 95% confidence interval (dotted lines) in each plot. See text for details.

In order to determine whether the opposite effects of NBQX and PPPA on reward was related to the site of injection within the VTA, we first correlate the changes in reward threshold produced by each drug. The correlation was calculated from the average percent change in reward threshold of the first three measures (between 20 and 54 min), a time at which PPPA and NBQX produced significant behavioral effects; they were transformed in absolute log unit deviation from baseline. Scatter plot presented in Figure 6 (top panel) shows that the magnitude of the attenuation effect of NBQX on reward was inversely related to the magnitude of the reward enhancement produced by PPPA. This inverse relationship seems to be due to the site of injection over the antero-posterior axis. In effect, the effectiveness of NBQX at attenuating reward is inversely related to the VTA antero-posterior level, being most effective in the anterior VTA (middle panel). The opposite was found with PPPA, and the more posterior was the injection the larger the reward enhancement (bottom panel).

## Discussion

A major finding of this study is that blockade of VTA AMPA receptors following *in situ* microinjections of NBQX, a selective AMPA receptor antagonist (Sheardown et al., 1990), attenuated in a dose-dependent manner reward induced by electrical stimulation of the medial posterior mesencephalon. The reward attenuation reflected by a rightward-shift of the R/F curve was not accompanied by a significant change in maximum rate of responding. This selective displacement of the R/F curve on the stimulation frequency axis demonstrates that NBQX produced a specific attenuation of the reward signal initiated by the electrical stimulation (Miliaressis et al., 1986).

The most likely hypothesis to account for the attenuation effect of NBQX is a reduction in glutamatergic excitatory inputs to dopamine neurons. Rewarding electrical stimulation increases dopamine cell firing and accumbens dopamine release (Moisan and Rompré, 1998; Hernandez and Shizgal, 2009). Specific reward attenuations are observed following systemic injection of low doses of the dopamine receptor antagonist, haloperidol (Benaliouad et al., 2007). Ventral midbrain microinjections of drugs known to increase and block dopamine impulse flow enhance and attenuate brain stimulation reward respectively (Rompré and Wise, 1989a,b; Wise and Rompré, 1989). Accumbens dopamine release induced by a cue associated with brain stimulation reward is blocked by a VTA microinjection of lidocaine, suggesting that it is mediated by synaptic activation of VTA neurons (Sombers et al., 2009). Glutamate containing terminals establish synaptic contacts with dopamine neurons (Carr and Sesack, 2000; Omelchenko et al., 2009) and brain stimulation reward is associated with an increase in VTA glutamate release an effect that is prevented by infusion of tetrodotoxin (You et al., 2001). Activation of glutamatergic efferents to dopamine neurons produces AMPA mediated EPSCs, increases dopamine cell firing and induces conditioned-place preference (Lammel et al., 2012). It thus appears likely that NBQX attenuated the reward signal initiated by posterior mesencephalic electrical stimulation, a signal carried by glutamatergic inputs to dopamine neurons. This hypothesis is rather inconsistent with previous findings showing that blockade of VTA AMPA receptors stimulates spontaneous motor activity and induces a conditioned-place preference (Harris and Aston-Jones, 2003; Harris et al., 2004; Nolan et al., 2010). It was also shown, however, that VTA microinjection of CNQX, an AMPA antagonist, also prevents morphine-induced increase in dopamine cell firing and morphine-induced conditioned-place preference (Harris et al., 2004; Jalabert et al., 2011). The valence of the motivational effect of AMPA receptor blockade may depend upon the level of activity of the dopamine system. Morphine, like brain stimulation reward, stimulates dopamine impulse flow and release and under such a condition of high dopamine activity blockade of AMPA receptors may reduce the positive reinforcing effect.

Electrophysiological studies have shown that activation of dopamine neurons by glutamate is also mediated by NMDA receptors. Electrical stimulation of VTA afferents to dopamine neurons, for instance, produces NMDA mediated EPSCs (see Ungless et al., 2001) and NMDA produces a dose-dependent increase dopamine cell firing (Seutin et al., 1990; Wang and French, 1993). Deletion of NMDA receptor from dopamine neurons abolishes burst firing, a mode of activity that is associated with enhanced dopamine release and reward (Gonon, 1988; Schultz, 2007; Zweifel et al., 2009). On the basis of these findings, microinjections of the selective NMDA antagonist, PPPA, was expected to attenuate reward. However, PPPA produced an enhancement rather than an attenuation of the reward signal initiated by posterior mesencephalic electrical stimulation. The reward enhancement was unrelated to the increase in maximum rate of responding since these PPPA effects occurred over a different time course. The effects of PPPA are very similar to those reported previously with the same dose by Bergeron and Rompré, (2013). To explain this unexpected enhancement of reward and operant responding, Bergeron and Rompré proposed that PPPA acted on NMDA receptors that were mainly composed of GluN2A sub-units and that these NMDA receptors were expressed on non-dopamine neurons. There is no direct evidence that dopamine neurons expressed NMDA receptors that are devoid of GluN2A receptors. However, GluN2A subunits are present in the VTA and some electrophysiological data suggest that they are expressed by non-dopamine neurons (Jones and Gibb, 2005; Suarez et al., 2010).

It is well established that exposure to drug reward induces synaptic plasticity at glutamatergic synaptic inputs to dopamine neurons; this plasticity is characterized by enhanced AMPA EPSCs, due to replacement of GluR2 sub-units for the more conductive GluR1 sub-units, and by reduced NMDA EPSCs (Ungless et al., 2001). Following these synaptic changes, the contribution of AMPA receptor to activation of dopamine neurons is stronger than that of NMDA and under this condition it is expected that blockade of AMPA receptors has a larger impact than blockade of NMDA receptors on the excitatory signal to dopamine neurons. That could explain why NBQX but not PPPA attenuated reward. For this hypothesis to be right, however, one has to postulate that brain stimulation reward induces an increase in AMPA/NMDA EPSC ratio at glutamatergic synapses on dopamine neurons. This might be the case as Boye et al., (2007) have shown that training for brain stimulation reward sensitizes to amphetamine induced forward locomotion, a sensitization effect that is associated with an enhanced AMPA/NMDA ratio (Saal et al., 2003). Carlezon et al., (2001), however, reported a reduction in VTA AMPA receptor sub-unit, GluR1, in animals that were trained to respond for brain stimulation reward, a finding inconsistent with an increase in AMPA/NMDA EPSC ratio.

A large amount of non-dopamine neurons are GABAergic neurons that provide a tonic inhibitory input to dopamine (Grace et al., 2007). Glutamatergic terminals establish synaptic contacts with non-dopamine neurons (Omelchenko et al., 2009) and these are stimulated by activation of NMDA receptors (Wang and French, 1995). It could be then that PPPA enhanced reward by removing the tonic GABAergic inhibition to dopamine neurons, as proposed by Bergeron and Rompré, (2013). Since the activity of VTA non-dopamine neurons is also stimulated by activation of AMPA receptors (Wang and French, 1995), it is not clear why NBQX did not produce effects that were similar to those of PPPA. A hypothesis that may reconcile the opposite effects is that NBQX and PPPA acted on different sub-populations of VTA neurons. Our correlation analyses tend to support this hypothesis. First we found that the magnitude of the reward attenuation effect of NBQX was inversely related to the magnitude of the reward enhancing effect of PPPA. This means that at sites where NBQX produced the larger reward attenuations, PPPA produced the smallest reward enhancements. A second analysis revealed opposite correlations between the anterior-posterior VTA level and the absolute change in reward produced by each antagonist. NBQX was most effective when injected in the anterior VTA while PPPA was most effective when injected in the posterior VTA. GABAergic neurons in the RMtg/tVTA receive a dense glutamatergic innervation from the lateral habenula and these provide an inhibitory input to VTA dopamine neurons (Brinschwitz et al., 2010). Selective activation of lateral habenula glutamatergic pathway induces EPSCs in RMtg/tVTA GABAergic neurons and induced conditioned-place aversion (Lammel et al., 2012). Dopamine cell firing is, respectively, reduced and enhanced by activation and inhibition of RMtg/tVTA neurons (Bourdy and Barrot, 2012). Rats self-administer NMDA antagonists into the VTA and the most effective sites are within or near the RMtg/tVTA (David et al., 1998; Webb et al., 2012). It is possible that PPPA enhanced reward by blocking some glutamatergic inputs to RMtg/tVTA neurons. Again, the reason why NBQX was not as effective as PPPA is unclear, particularly because EPSCs induced in RMtg/tVTA neurons by stimulation of glutamatergic inputs are mediated, at least in part, by AMPA receptors (Lecca et al., 2011). It could be that PPPA enhanced reward by acting on RMtg/tVTA and on other VTA neurons since it is still effective when injected in more VTA anterior sites; this conclusion was proposed by Bergeron and Rompré, (2013) on the bases of the results that they obtained with another NMDA antagonist, R-CPP.

Shabat-Simon et al., (2008) have shown that the rewarding effect of opiates as measured with the conditioned-place preference and self-administration paradigms is prevented by blockade of VTA AMPA receptors. Interestingly, they showed that CNQX was most and least effective when injected, respectively, into the anterior and posterior VTA. These findings parallel the results that we obtained in the present study with NBQX.

Another observation suggesting that NBQX and PPPA acted on different sub-population of VTA neurons to alter brain stimulation reward is that PPPA, but not NBQX, produced a significant increase in maximum rate of responding. The fact that the enhancement of reward and operant responding occurred over a different time course suggests that they are mediated by the action of PPPA on different substrates. A similar dissociation between changes in reward and operant responding has been reported previously with different pharmacological treatments (Rompré, 1995; Boye and Rompré, 2000; Benaliouad et al., 2009; Gallo et al., 2010). That reinforces the hypothesis that some VTA neurons are more sensitive to NMDA than AMPA receptor blockade and vice versa. Investigation of NMDA and AMPA synaptic responses on different VTA neuron sub-populations in animals previously trained for brain stimulation reward may provide important insights into the identification of the neural substrates of reward.

## Author contributions

Pierre-Paul Rompré, Charles Ducrot and Emmanuel Fortier participated to the design of the experiment. Charles Ducrot, Emmanuel Fortier and Claude Bouchard performed the surgery, the behavioral tests and the analysis the data. All contributed to the final version of the manuscript.

### Conflict of interest statement

The authors declare that the research was conducted in the absence of any commercial or financial relationships that could be construed as a potential conflict of interest.
